# Multi-Target Mechanisms of Phytochemicals in Alzheimer’s Disease: Effects on Oxidative Stress, Neuroinflammation and Protein Aggregation

**DOI:** 10.3390/jpm12091515

**Published:** 2022-09-15

**Authors:** Javad Sharifi-Rad, Simona Rapposelli, Simona Sestito, Jesús Herrera-Bravo, Alejandra Arancibia-Diaz, Luis A. Salazar, Balakyz Yeskaliyeva, Ahmet Beyatli, Gerardo Leyva-Gómez, Carlos González-Contreras, Eda Sönmez Gürer, Miquel Martorell, Daniela Calina

**Affiliations:** 1 Facultad de Medicina, Universidad del Azuay, Cuenca 14-008, Ecuador; 2 Department of Pharmacy, University of Pisa, Via Bonanno 6, 56126 Pisa, Italy; 3 Department of Chemistry and Pharmacy, University of Sassari, Via Vienna 2, 07100 Sassari, Italy; 4 Departamento de Ciencias Básicas, Facultad de Ciencias, Universidad Santo Tomas, Talca 3460000, Chile; 5 Center of Molecular Biology and Pharmacogenetics, Scientific and Technological Bioresource Nucleus, Universidad de La Frontera, Temuco 4811230, Chile; 6 School of Biochemical Engineering, Pontificia Universidad Catolica de Valparaiso, Av. Brasil 2085, Valparaiso 2362803, Chile; 7 Faculty of Chemistry and Chemical Technology, Al-Farabi Kazakh National University, Almaty 050040, Kazakhstan; 8 Department of Medicinal and Aromatic Plants, University of Health Sciences, Istanbul 34668, Turkey; 9 Departamento de Farmacia, Facultad de Química, Universidad Nacional Autónoma de México, Ciudad de México 04510, Mexico; 10 Department of Nutrition and Dietetics, Faculty of Pharmacy, and Centre for Healthy Living, University of Concepción, Concepción 4070386, Chile; 11 Department of Pharmacognosy, Faculty of Pharmacy, Sivas Cumhuriyet University, Sivas 58140, Turkey; 12 Department of Clinical Pharmacy, University of Medicine and Pharmacy of Craiova, 200349 Craiova, Romania

**Keywords:** Alzheimer’s disease, phytochemicals, neuroinflammation, oxidative damage, protein aggregation, molecular mechanisms, neuroprotective effects

## Abstract

Alzheimer’s disease (AD) is a neurodegenerative disease characterized by a tangle-shaped accumulation of beta-amyloid peptide fragments and Tau protein in brain neurons. The pathophysiological mechanism involves the presence of Aβ-amyloid peptide, Tau protein, oxidative stress, and an exacerbated neuro-inflammatory response. This review aims to offer an updated compendium of the most recent and promising advances in AD treatment through the administration of phytochemicals. The literature survey was carried out by electronic search in the following specialized databases PubMed/Medline, Embase, TRIP database, Google Scholar, Wiley, and Web of Science regarding published works that included molecular mechanisms and signaling pathways targeted by phytochemicals in various experimental models of Alzheimer’s disease in vitro and in vivo. The results of the studies showed that the use of phytochemicals against AD has gained relevance due to their antioxidant, anti-neuroinflammatory, anti-amyloid, and anti-hyperphosphorylation properties of Tau protein. Some bioactive compounds from plants have been shown to have the ability to prevent and stop the progression of Alzheimer’s.

## 1. Introduction

In 1906, Alois Alzheimer described the first histopathological case of presenile dementia of the 51-year-old patient Auguste D. with amyloid plaques and neurofibrillary tangles (NFTs) in the upper cortical layers, a new era of medicine that would show us the temporary vulnerability of people. In 1910, Emil Kraepelin assigned the eponymous Alzheimer’s disease (AD) to the pathology characterized by Alois Alzheimer. Symptoms of AD comprise a syndrome of progressive cognitive and functional decline. The early stage includes short-term memory failure (recent conversations, names, or events), apathy, and depression. Later symptoms include disorientation in time and space, confusion, impaired communication, difficulty in personal hygiene, loss of speech, movement, and difficulty swallowing. AD is characterized by the accumulation of the protein fragment beta-amyloid in plaques outside the brain neurons and twisted strands of the protein tau in the form of tangles inside neurons. Due to the protein encrustations, there is a persistent inflammatory process, brain decrease in glucose metabolism, and progressive neuronal death [1,2]. The increase in life expectancy is one of the factors that has led to an increase in the number of AD cases worldwide [3,4]. Regarding pharmacological intervention, the few anti-AD therapies currently available on the market are unable to stop the onset and the progression of the disease. They provide modest but meaningful benefits since they help in mitigating symptoms, reducing clinical progression, and delaying disability [5]. These drugs are defined as “symptomatic” and are especially useful in the first phase of the disease or cases of mild or moderate forms. The U.S. Food and Drug Administration (FDA) has approved a few drugs for Alzheimer’s treatment: rivastigmine, galantamine, donepezil, memantine, and memantine combined with donepezil. The approved drugs, except memantine, have acetylcholinesterase inhibitors (AChEI), so they increase the presence of acetylcholine (ACh) that favors the modulation of the synaptic process. Memantine is an antagonist of the NMDA receptor subtype of glutamate receptor and reduces the excitotoxic effects of glutamate release [1,6]. The unbalance of oxidative stress (OS) and a chronic inflammatory process requires the timely participation of new treatments that exhibit greater efficacy in AD and fewer adverse effects.

The epidemiological projections of AD and the deficiencies that appear in current drugs for this pathology raise the need to explore new sources of compounds with effective protective effects against the acute neurological deterioration caused by AD. Among the challenges of this research is to identify effective, preventive, and curative neuroprotective bioactive components, whilst being well tolerated and free from adverse side effects to patients. With this background, natural compounds emerge as attractive complementary and alternative options for the management of neurodegenerative diseases [7]. Phytochemicals are bioactive secondary metabolites that include alkaloids, polyphenols, terpenoids, organosulfur compounds, limonoids, furyl compounds, polyenes, thiophenes, peptides, saponins, sterols, lignans, tannins, and stilbenes [8,9,10]. According to the World Health Organization, approximately 80% of the world’s population depends on phytochemicals or products of medicinal plants [11].

This article presents the current state of Alzheimer’s research, the pharmacological proposals, and the various studies with phytochemicals, from the point of view analytical and critical, highlighting the advantages and possible limitations. The nanoformulations section offers a perspective on the impact of new formulations and the advent of new technologies to aid phytochemical treatment.

## 2. Review Methodology

The eligible studies published on the molecular mechanisms of action with neuroprotective effect of phytochemicals were searched in the following databases PubMed/Medline, Embase, TRIP database, Google Scholar, Wiley, and Web of Science using the following MeSH terms: “Acetylcholinesterase”, “Aged”, “Anti-Inflammatory Agents /therapeutic use”, “Amyloid beta-Peptide/metabolism”, “Alzheimer Disease/etiology”, “Alzheimer Disease/diagnosis”, “Alzheimer Disease/drug therapy”, “Alzheimer Disease/metabolism”, “Dietary Supplements”, “Neurofibrillary Tangles”, “Disease Progression”, “Disease Management”, “Plants”, “Medicinal/chemistry”, “Phytochemicals/chemistry”, “Phytochemicals/pharmacokinetics”, “Phytochemicals/therapeutic use”.

Inclusion criteria: preclinical studies on cell lines or AD models in laboratory animals, treatments with phytochemicals (bioactive compounds and essential oils), results supported by histopathological and behavioral evidence, and articles written in English.

Exclusion criteria: summaries of incomplete studies and presentations at conferences, duplicate articles, experiments that had associated homeopathic preparations, and experiments without a control group.

The scientific names of the plants have been validated with WorldFlora online and the chemical formula with ChemSpider [12,13]. The most representative data regarding neuroprotective mechanisms of phytochemicals in AD have been summarized in an image and tables.

## 3. The Pathogenesis of AD: A Brief Overview

Since its discovery almost a century ago, a huge advancement in identifying the main features of the pathology and the potential triggering factors has been accomplished, especially in the last 30 years. Even though the ultimate nature of AD remains obscure, several factors and different “hypotheses” have been formulated in an attempt to unravel AD etiopathology [14].

The cholinergic hypothesis argues that the reduction of learning and mnemonic processes at the base of the disease are due to low levels of ACh in neurons; reduced ACh, is associated with an insufficient synthetic action of choline O-acetyltransferase (ChAt) and a high catalytic action of acetylcholinesterase (AChE), finally, lead to the loss of cholinergic transmission at the pre-synaptic level [15]. ACh is a neurotransmitter of the CNS and peripheral nervous system (SNP) and is generally synthesized in neurons by the enzyme ChAt starting from choline and Acetyl-CoA as a source of the acetyl group. The hydrolytic degradation of ACh is carried out by esterases which convert it back into choline and acetic acid blocking the transmission of the cholinergic signal: in particular, AChE acts to a greater extent in the CNS and are responsible for most of ACh degradation. Butyrylcholinesterase (BuChE) plays a secondary role, and it has been shown that in some areas of the brain, the increased level is generically linked to a decrease in AChE levels. BuChE is mainly expressed in the liver and plasma and shows a much lower affinity for ACh than AChE. Consequently, both enzymes are useful therapeutic targets for the treatment of AD [16]. Cholinergic dysfunction was the first hypothesis postulated and is considered one of the most relevant causes of AD. Coherently, almost all the pharmacological options currently in therapy are aimed at strengthening the cholinergic system through the inhibition of the AChE/BuChE enzymatic function [17].

Later, in-depth investigations of the AD brain revealed the presence of abnormal protein aggregates, the so-called amyloid hypothesis. Currently, the neurotoxic senile plaques represent the most relevant histopathological characteristics of AD. Amyloid plaques are extracellular deposits of the amyloid beta (Aβ) protein, which is generated from sequential proteolytic cleavage of its precursor called APP (amyloid precursor protein). APP is a transmembrane protein involved in synaptic plasticity, brain development, and memory. APP metabolism takes place thanks to three enzymes called Aβ-amyloid precursor protein-cleaving enzyme (BACE), β-BACE, and γ-secretases, and it follows two different transformation routes, known as the amyloidogenic and non-amyloidogenic pathways. In the non-amyloidogenic pathway, which is the predominant one, APP is cleaved by α-secretase producing two by-products: a soluble fragment (sAPPα), which is released, and the CTF83 fragment, which remains associated with the membrane. γ-Secretase cleaves APP in the region of the Aβ peptide, thus preventing the formation of senile plaque and thus exerting a neuroprotective action. Subsequently, the CTF83 fragment is, in turn, cleaved by the γ-secretase to form a further Aβ fragment_17–40/42_; alternatively, the sAPPα fragment can be cleaved by the β-secretase to form the Aβ _1-16_ fragment. In the amyloidogenic pathway, APP undergoes cleavage by BACE-1, which produces both the sAPPβ and the CTF99; while the last remains associated with the membrane and then is cleaved by γ-secretase, sAPPβ is released and mainly converted into the neurotoxic Aβ_1-40/42_ peptide, the main constituent of the Aβ agglomerates [18].

Another relevant pathological marker in AD is the presence of intracellular NFTs. NTFs contain, as their first component, aggregates of the protein tau (τ) in a hyperphosphorylated state. The tau protein is a filamentous protein associated with microtubules; in healthy neuronal cells, following consecutive and reversible enzymatic processes of phosphorylation and dephosphorylation, it has the task of assembling and stabilizing microtubules and determining their binding with other components of the cytoskeleton [19,20]. In pathological conditions, however, the excessive hyperphosphorylation by specific kinases and phosphatases seems to lead to conformational changes of the protein itself, which not only prevents the stabilization of microtubules but also leads to the formation of NFTs. These insoluble aggregates envelop the nucleus of the neuronal cell, causing neuronal death and, ultimately, cognitive dementia [18].

Protein agglomerates (senile plaques and NFT), characteristic of the disease, are recognized as a threat to the immune system, which triggers the inflammation process. In this context, microglia, the cell system that supports and protects neurons, and astrocytes, play a key role in maintaining synaptic integrity and exert a double role in the inflammatory process. Both systems release pro-inflammatory mediators, such as cytokines, chemokines, reactive oxygen species (ROS), and complement proteins, thus promoting a neuroprotective function thanks to cytotoxic and phagocytic activity. However, increased activation of these systems could result in neuronal damage [21].

It was also observed that microglia can bind Aβ plaques via Toll-like receptors (TLRs) or receptors for advanced glycation end products (RAGE), both expressed on the cell surface. This process promotes microglia activation, which in turn causes a greater secretion of pro-inflammatory cytokines, chemokines, and ROS, as well. These factors contribute to the stimulation of astrocytes, whose activation amplifies the pro-inflammatory signals [22]. The presence of activated microglia would seem at first to exert a phagocytic response towards the plaques, mediated by the release of the insulin-degrading enzyme (IDE), and then a neuroprotective action [23]. In pathological conditions, the protracted inflammatory condition led to a disproportionate release of pro-inflammatory factors and to an increase of OS, which reverses the neuroprotection into neurotoxicity. Therefore, the hyperactivation of both inflammatory processes and immune response has an ambiguous cause/effect role and its alteration could constitute a self-defence mechanism useful in slowing the pathology.

Among the factors potentially involved in the pathophysiology of AD, it must be mentioned also metallostasis, i.e., the alteration of the homeostatic levels of metals. In the human brain, metal ions such as copper (Cu), zinc (Zn), and iron (Fe) are involved in numerous metabolic and enzymatic processes and their concentrations are finely regulated under normal physiological conditions. In the brains of AD patients, a very high level of these ions was instead observed, thus giving rise to the “metal ion hypothesis”. Abnormal accumulation or altered trafficking of bio-metals, in particular Cu, could lead to direct or indirect cellular damage. Metals can induce injury to biostructures, such as proteins, interacting with some specific structural regions and destabilizing their native conformation; coherently, many studies revealed that metals could accelerate the cytotoxicity of misfolded protein aggregates. However, metallostasis can increase ROS production leading to an enhanced level of toxic radicals [24]. Finally, OS is rising prominent attention in neurodegenerative research. The brain, as high metabolically active organ, generates a high amount of ROS [25]. Although ROS are normal metabolic by-products and possess a cellular messaging function, they are intrinsically dangerous due to the presence of an unpaired, highly reactive electron, able to react with different cellular macromolecules thus altering their functions. Cells have developed enzymatic antioxidant systems such as superoxide dismutase (SOD), catalase, and glutathione peroxidase to keep ROS at physiological concentrations. An imbalance between ROS production and antioxidant defence leads to OS and cellular damage. Several studies have shown that OS is deeply implicated in the pathogenesis and aetiology of AD. Aβ plaques, NTFs, dysregulated metallostasis, and abnormal neuroinflammation could contribute to increased ROS levels, leading to neuronal damage, but OS is also deeply involved in the rise and progression of these mechanisms, thus creating a self-feeding vicious cycle. Either cause or consequence, OS could be defined as a *fil rouge* connecting all the AD hallmarks already described [26].

AD is a complex and multi-factorial disease, and both the diagnosis and the treatment require careful analysis. A comprehensive evaluation integrates pathological AD biomarkers (cerebrospinal fluid [CSF] and amyloid PET), but the clinical diagnosis requires the characterization of symptoms and level of impairment into a profile via a clinical evaluation. Moreover, timely detection is crucial to avoid potentially harmful delays in receiving appropriate care, therefore symptoms should not be overlooked [27].

## 4. Current Pharmacotherapeutic Trends in the Management of AD

Based on clinical evidence, different stages have been identified and used to classify the progress of the disease. At the very beginning, the patients present Mild cognitive impairment (MCI), a condition characterised by memory impairment with relative sparing of other cognitive domains. MCI could be considered a prodromal phase, representing a transitional condition between healthy aging and very mild AD [28]. The following Early stage is mainly characterized by small short-term memory deficits and difficulty in remembering details (e.g., forgetfulness, losing track of the time, becoming lost in familiar places). The onset of these symptoms is gradual, and they are often attributed to aging and stress, therefore this stage is quite ignored. As dementia moves on to the Middle stage, a progressive deterioration of memory occurs increasing difficulties in language and worsening the coordination of motor sequences, resulting in the patient’s loss of independence. The rise of the Late stage is characterised by a growing impact on movement and physical capabilities, the first psychotic symptoms and a complete loss of language: the patient is no longer autonomous [14].

During the illness, most patients will experience neuropsychiatric symptoms or problem behaviours, such as behavioural and psychological symptoms of dementia (BPSD). BPDS are characterized by alterations of perception (hallucinations, misidentification), thinking (delusions), mood (depression, anxiety, apathy), and behaviour (aggression, agitation, disinhibition, wandering, socially or sexually inappropriate behaviour) [29]. BPSD are highly prevalent in all types of dementia and may occur in MCI and all stages of AD patients; since now, no agreement on the possible relationship with disease evolution has been reached. What is certain is that BPSD is associated with more rapid decline, worse quality of life, higher distress, earlier institutionalization, and greater health care employment and costs; therefore, the BPSD should be particularly monitored, and their management should include the following steps: symptoms evaluation, identification, and treatment of potential biological factors or triggering events, non-pharmacological treatments, pharmacological therapy, complementary and adjuvant medicine, educational, and psychological support for the caregiver [30].

### 4.1. Non-Pharmacological Interventions

The non-pharmacological treatments (NPT) are frequently chosen as a first-line option to mitigate AD symptoms and represent the most valid alternative and/or complement to pharmacological treatment [3,31]. NPTs improve patients’ Quality of Life (QoL), reduce the stress of caregivers, and ameliorate the environment; they include a wide range of approaches and techniques such as exercise and motor rehabilitation, cognitive intervention, occupational therapy, and psychological therapy, often mixed and combined. Also, the application of new technology (ICT, assistive devices and domotics, virtual reality and gaming, telemedicine) could offer additional support to AD treatments [30].

### 4.2. Pharmacological Interventions

Unfortunately, there are no curative therapies for AD and other common etiologies of dementia. Current pharmacological therapies aim to delay the progression of neurocognitive and physical decline; however, they do not stop the damage and destruction of neurons. FDA-approved pharmacological treatments for AD can be divided into two classes: AChEIs and NMDA receptor antagonists. The former is prescribed to improve cognitive symptoms (memory and attention) and behavioural symptoms (apathy and agitation), but their effectiveness decrease as the disease worsens. AChEIs facilitate central cholinergic activity by preventing the degradation of ACh by AchE, thus increasing its concentration in the synaptic cleft and promoting its interaction with pre- and post-synaptic receptors. This class include donepezil and rivastigmine, approved and labelled for mild, moderate, and severe AD dementia, and galantamine, administered in mild and moderate AD. Among the AChEI, it is worth mentioning tacrine, one of the first drugs for the treatment of AD approved by the FDA, was later abandoned due to its toxicity. All the AChEI have a low affinity for peripheral receptors but are lipophilic enough to pass the blood-brain barrier and exert their action in the CNS. In addition to AChE inhibition, Donepezil and Rivastigmine also inhibit BuChE activity, while Galantamine acts as an allosteric modulator of ACh nicotinic receptors. Despite the diverse additional mechanism of action, no high-quality data evidenced significant differences in their efficacy [27].

The second class of drugs includes Memantine, a cleared low-to-moderate affinity NMDA-receptor channel blocker. Its application is based on the evidence that the brain of AD patients presents over-stimulation of this receptor, responsible for releasing glutamate which tends to trigger neuronal toxicity. Memantine was approved by FDA in 2002 for moderate to severe AD dementia, both as monotherapy and in combination with a ChEI (often added to an existing ChEI treatment). Recently, Aducanumab, an anti-Aβ monoclonal antibody has been approved by FDA (June 2021) as an anti-AD treatment. Aducanumab therapy appears to be able to slow the course of the disease in its early stages. Although this drug has been approved by the FDA, its clinical effectiveness is still controversial [32].

#### 4.2.1. Pros and Cons of AChEIs

The current pharmacological management of AD is restricted to the drugs approved for use by the FDA. Three correspond to the AChEI: rivastigmine, donepezil and galantamine, and the remaining to memantine, the NMDA antagonist [33,34].

AChEI is suggested to stop and/or decrease the action of the cholinesterase enzyme in the synaptic cleft of cholinergic cells responsible for the catalytic action of the neurotransmitter ACh hydrolysis in patients with AD [33]. Its pharmacological action points to an improvement in central cholinergic neurotransmission, reducing cognitive deterioration. All three drugs delay neurodegeneration and stabilise cognition when used in patients with mild to severe AD symptoms, as shown in clinical trials. The highest doses reported in patients with advanced disease are donepezil 10 and 23 mg tablets and rivastigmine patches 13.3 mg/24 h. Despite the efficacy of AChEI in AD, reports indicate that between 5 and 20% of patients show adverse effects mainly associated with the digestive, cardiac and pulmonary systems, having to generate personalized tolerance surveillance in patients depending on the maximum recommended dose and safety versus dosage [33,35,36]. These disadvantages are increased mainly because this type of neurogenerative disease is accompanied by other pathologies typical of geriatric patients such as hypertension, hypercholesterolemia, and diabetes, among others. Other limitations presented by AChEI is that the action of restoring cholinergic neuronal transmission has been observed with a maximum in patients tolerant to the drug for 3 years and its efficacy is in symptomatic patients modulating a specific action of the neural network, being mere stabilizers of the CNS, but its contribution is very insignificant in modifying properties that trigger AD, this is due to the multifactorial conditions of this neurodegenerative pathology in which pharmacology that points to a broader spectrum of action could generate greater benefits [37].

Memantine, the antagonist of NMDA, is focused on reducing the overexcitation of glutamate receptors, which is expressed proportionally to the progression of the pathology, thus, allowing its cognition functional integrity [35,38]. This medicine is used as monotherapy or in add-on therapy with AChEI. Monotherapy has shown beneficial effects in the short and long term in patients with moderate to severe AD, improving their quality of life, cognition, and behavioral and psychological symptoms of neurodegenerative diseases [33,34], It is normally well-tolerated without the adverse effects presented by the AChEI at the gastrointestinal level, which are the most severe; however, some patients have manifested negative effects such as dizziness, constipation, headache, cough, and drowsiness [33,35,36]. The dosage ranges from 10 g to 28 mg/day depending on the progression of the disease [34,35,36]. In complementary therapies with AChEI, its beneficial effects are observed, producing a decline in AD; however, it has been reported that this condition is visible within a maximum period of 3 years, after which AD continues to progress [37].

Challenges in current AD treatment therapies are focused on reducing adverse effects and increasing drug action time in terms of neurodegeneration decline effectiveness. One of the main keys is the integrative and non-specific therapy design, given the multifactorial mechanisms that trigger AD. This implies, promoting new AChEI or NMDA receptor modulators since they continue to be neuron-specific therapies, aiming only at the arrest of one of the pathophysiological processes triggered.

#### 4.2.2. Drug Repurposing in AD

An ideal anti-AD therapy should block the neurodegenerative process from its roots, whereas the currently available treatments offer benefits just in the management of symptomatology. Despite the intense efforts accomplished, no resolutive therapy for AD has been yet identified, pushing both pharma and academia in finding more effective therapeutic modalities. The wide scientific progression in the last century allowed researchers to investigate deeper the mechanism of action of old/existing/available drugs, revealing additional pharmacological activities and expanding their therapeutic potential [39].

Drug repurposing offers different advantages such as reaching wider markets and greater numbers of patients and bypassing much of the exploratory work needed to identify new molecules, new mechanisms of action, formulation, manufacturing procedures and pharmacokinetics. Finally, market-tested drugs have already passed clinical trials and have proven their safety and efficacy for their primary application, allowing for drastically reducing the time for new market approval [40]. Nowadays, drug repurposing is a common approach to AD drug development and represents an important percent of trials in the current AD pipeline. A recent study assessing the repurposed AD therapies represented in Phase I, II, and III since February 2020 identified 53 clinical trials investigating 58 total repurposed drugs with the various initial clinical application (20% hematologic-oncologic, 18% cardiovascular, 14% psychiatric, 12% diabetes, 10% are neurologic, 26% other conditions) [41].

#### 4.2.3. Candidate Vaccines in AD

To treat Alzheimer’s disease, vaccines as new therapeutic trends and promising research have been developed by researchers in recent years. The development of a new type of vaccine in various diseases is difficult, expensive, and requires special and time-consuming techniques [42,43,44]. One technique that helps streamline the process is repurposing existing vaccines or drugs since therapies approved for use in humans have already passed the barrier of proving their safety [45,46,47]. Regarding Alzheimer’s disease, AD vaccine development interventions consist of repurposed therapies [48]. The most common aspects of AD involve the accumulation of beta-amyloid plaques, tau proteins, and neuroinflammation; these are the main targets for candidate anti-Alzheimer vaccines [48,49,50]. The goal of developing AD vaccines is to reduce or prevent the progression of the disease. There are several approaches which researchers are using to develop Alzheimer’s vaccines. Some approaches target beta-amyloid plaques, while others focus on tau protein, and others are immunomodulatory [48] (Table 1).

#### 4.2.4. Other Interventional Approaches in AD: One Step Forward

One of the lessons learnt by the huge amount of research focused on AD and other neurodegenerative diseases is that these kinds of pathologies are based on a few etiological events intertwined with each other; therefore, a more holistic approach could contribute to the development of more resolutive therapies. For instance, the development of multi-target ligands (MTDLs), or compounds capable of interacting with more than one relevant target in the pathogenesis of the disease, is gaining growing relevance [57].

One of the most followed MTDL design strategies consists in combining different pharmacophores provided by specific biological activities, linked together by suitable linkers or directly fused/merged, into a single molecule. These molecules, designed to preserve their interaction with specific binding sites, potentially act on several fronts, allowing a more effective slowdown of the neurodegenerative process [57].

## 5. Natural Phytochemicals as New Potential Therapeutic Strategies in AD

Experimentally, the search for and obtention of bioactive compounds has been promoted and categorized under the name of nutraceuticals [58,59,60]. Stephen de Felice proposed this concept in 1989 and defined it as “any substance considered as food or as part of it that provides medical or health benefits, including the prevention or treatment of a disease” [61]. Various definitions for these compounds have been established by European and American regulatory governmental organizations [62]. There is a convergence in the definitions of nutraceuticals, establishing that these groups of substances can be chemically or biologically active and intrinsic constituents of matrices of natural origin from microalgae, fruits, vegetables, grains, medicinal herbs, and agro-industrial residues (leaves, peels, pulps inedible, seeds, low-quality fruits, pits, stems, skins), among others [63,64,65,66]. They are commonly presented in a non-food format (pills, powder, liquids, etc.) and administered in concentrated doses. As a result of a significant increase in their concentration, a positive modulating effect on human health has been presumed, thus considered a beneficial supplement for the care and maintenance of healthy individuals, due to the improvement of biological functions, as well as in the prevention of diseases and the complementary treatment for declared pathologies, as is the case of EA [62,67,68,69]. Various investigations have shown that nutraceuticals are capable of fulfilling neuroprotective functions on molecular mechanisms that trigger AD, acting as antioxidants or anti-neuroinflammatory drugs, taking effect on the aggregation of β amyloid peptides, the hyperphosphorylation of the Tau protein and the decrease of the neurotransmitter ACh in cholinergic neurons [70,71,72]; such evidence allows us to project a scenario where AD could have possible preventive treatments and/or have a slowing palliative effect in patients who already suffer from it [73,74,75].

Current trends aim toward the use of “smart drugs” [37,59,76], with complementary and adjuvant therapies, using phytopharmaceuticals with antioxidant properties of positive modulation on neuroprotection, covering a wide spectrum of targets that are triggered in AD, such as energy metabolism of the mitochondria, modulation of genes related to the amyloidogenic pathway, and interaction on Tau hyperphosphorylation, among others. Therapies with phytopharmaceuticals with known neuroprotective effects could generate neuroenhancers that may greatly improve cognitive capacity in patients with AD, reducing the main disadvantages of conventional pharmacology [31].

### 5.1. Naturally-Occurring Bioactives in AD

Many experimental preclinical pharmacological studies have demonstrated the efficacy of phytochemicals in the management of AD, and in recent decades, dozens of bioactive compounds and natural plant extracts have been used as potential agents against dementia and AD [77,78,79]. *Justicia adhatoda* L. (syn. *Adhatoda vasica* Nees), *Ferula assa-foetida* L. [80], *Amorphophallus paeoniifolius* (Dennst.) Nicolson (syn. *Amorphophallus campanulatus* Decne) [81], *Catharanthus roseus* (L.) G.Don [82], *Ginkgo biloba* L. [83], *Panax ginseng* C.A.Mey. [84], *Zingiber officinale* Roscoe [85], and the list go on.

Various essential (volatile) oils of different plants were used in combating AD, for example, *Coriandrum sativum* L. [86], *Zosima absinthifolia* Link [87], *Hedychium gardnerianum* Sheppard ex Ker Gawl. [88], Persicaria hydropiper (L.) Delarbre (syn. *Polygonum hydropiper* L.) [89], and *Lavandula* spp. [90]. Phenolic and flavonoid contents [91], catechins [92], coumarin [93], resveratrol [94], quercetin [95]; curcumin [96], ginkgolide [97], glycyrrhizin [98], monoterpenoid [99], and S-allylcysteine [100] which isolated from various medicinal plants have been identified to have the potency to prevent AD. Phytochemicals are used widely nowadays in green synthesis which is an ecofriendly process because of their reducing and stabilizing properties [101,102,103]. The green synthesis of silver, gold, and zinc oxide nanoparticles (NPs) using plant extracts such as *Lampranthus coccineus* (Haw.) N.E. Br. and *Malephora lutea* Schwantes [104], *Terminalia arjuna* (Roxb. ex DC.) Wight & Arn. [105] and *Sabal palmetto* (Walter) Lodd. ex Schult. & Schult.f. (syn. *Sabal blackburniana Schult. & Schult.f.*) [106], respectively, showed activities that can be useful in treating AD. Experimental evidence has shown that a large variety of bioactive molecules classified as phytochemicals can reduce and block molecular cascades involved in the progression of AD.

### 5.2. Role of Phytochemicals in AD: Underlying Molecular Mechanisms of Action

#### 5.2.1. Antioxidant Mechanisms

Although AD has complex multifactorial, effects OS is key to the initiation of the pathophysiological process in cholinergic neurons [107,108,109]. OS refers to the excessive oxidation of molecules at the intracellular level, resulting in damage and leading to death processes in the cell. This physiological phenomenon is created by compounds called ROS, such as superoxide radicals, hydrogen peroxide (H_2_O_2_), hydroxyl radicals, nitric oxide and their metabolites. The reactivity and harmfulness of these ROS vary within the cell [110]. The greatest negative impact is formed during oxidative phosphorylation in the mitochondrial crests at the end of the aerobic respiration process [111,112,113,114]. Oxidation processes can affect DNA, causing nucleotide dimerization and causing errors in replication processes. Also, a functional alteration in essential structural or catalytic proteins can be generated [115]. Finally, they have an impact on the unsaturated fatty acids chain of membrane phospholipids, causing cell lysis [116,117,118]. This OS is buffered in the cell using “antioxidants”, a concept coined for those expressions that counteract the negative effects of this type of disturbance at the intracellular level [119,120]. The antioxidant’s mode of action on this OS is categorized according to their ability to:

(1) inhibit the formation of free radicals (indirect antioxidants),
(2) directly eliminate chemically generated free radicals (direct antioxidants)
(3) strengthen the cellular capacity to cope with high ROS loads, enzymatically detoxify accumulated ROS or promote the damage repair caused by oxidation (metabolic antioxidants) [116].

It has been proposed that most bioactive molecules act as direct antioxidants and they do not have a dependence on endogenous intracellular enzymes to exert their primary action by directly reacting with free radicals [69,121]. At the systemic level in the human organism, the nervous system seems to be the one with the greatest vulnerability to ROS, compared to other organs and tissues, mainly due to the high rate of oxygen consumption, limited endogenous antioxidant capacity, high content of steroidal lipids, and high concentrations of metallic catalysts, which favors a constant oxidative physiological condition at the cerebral cortex level [111,122,123].

ROS have been postulated to be the main cause of neurological deterioration, triggering pathologies such as AD, Parkinson’s disease (PD), and Huntington’s disease (HD) [72,116,124]. Particularly for AD, excessive OS manifests itself from the early stages of the disease [72,125]. Hydrogen peroxide seems to be the relevant actor in promoting excessive oxidation in neuronal cells. Under normal physiological conditions, peroxisomes are responsible for enzymatically controlling superoxide radicals and H_2_O_2_ thanks to catalase and peroxidase. However, when the hyperphosphorylated Tau protein is overexpressed (which is a pathophysiological indicator of AD) a depletion of these organelles has been evidenced, suggesting it is the cause of H_2_O_2_ and ROS increase [72,108,126]. Kou et al. [127] evidenced heterogeneity in the peroxisomal distribution in neurons of patients with AD, which was concentrated in the soma and absent in the dendrites, implying that Tau does not allow the correct transit of these organelles, being an abnormally phosphorylated cytoskeleton protein, the possible cause of the increase in the oxidizing environment. On the other hand, the existence of a link between the increase in ROS, H_2_O_2_, metal ions, and the peptide Aβ [125] has been suggested. Hydrogen peroxide is one of the most accepted AD inducer molecules, composed of 40 to 42 amino acids, and can form neurotoxic aggregates that ultimately cause neuronal death and consequently cognitive deterioration [124,128,129,130]. Peptides (Aβ) have a high affinity for Cu ions, producing a 1:1 complex, which favors the aggregation and formation of amyloid plaques. In this reaction, H_2_O_2_ and the increase in ROS are linked, thus evidencing the key role of the H_2_O_2_ molecule in the development of AD pathology [125].

Various in vitro models carried out with neurons isolated from the CNS and/or with immortalized cell lines, demonstrated that H_2_O_2_ causes cell death through apoptosis [118,131,132,133,134,135]. The human neuroblastoma SH-SY5Y is among the most used cell lines [131,136,137,138,139,140,141,142], a subclone of a parental neuronal line SK-N-SH, which originated in 1970 from a biopsy metastatic tumor. Their most important characteristics are their ability to have a mixed culture (adherent and in suspension) and to differentiate into specialized neurons through markers, allowing adrenergic, cholinergic, and dopaminergic models [142,143], to be a basic model for the study of neurodegenerative pathologies, such as AD.

Recently, Angeloni et al. [144] evaluated the antioxidant and anti-inflammatory capacities of aqueous, methanolic and ethanolic extracts of coffee grounds (CG) on SH-SY5Y cells. A significant increase in cell viability was observed when a neuroblastoma culture was subjected to OS using H_2_O_2_ and subsequently treated with various types of CG extracts. However, the authors concluded that a greater study of extracts constituents is necessary for the recognition of possible bioactive molecules with neuroprotective capacity. It is suggested that the fraction of phenolic compounds, consisting mainly of caffeic acid (CA), quinic acid (QA), and the chlorogenic acids family, are the main compounds responsible for these properties [145,146,147,148,149,150,151]. Gao et al. [152] demonstrated that chlorogenic acids treatment significantly inhibited autophagy caused by Aβ25-35 by modulating lysosomal function in SH-SY5Y cells, thereby attenuating the loss of CA1 neurons and cognitive defects in APP/PS1 mice, evidencing the neuroprotective potential of this compound against diseases such as AD. Kim et al. [153] evaluated the effects of H_2_O_2_-induced cell damage of two caffeoylquinic acids (CQA) derivatives obtained from *Dipsacus asper* Wall. ex C.B. Clarke plant extracts using the human neuroblastoma cell line SH-SY5Y. Their results showed attenuation in neuronal apoptosis due to a decrease in the activation of Caspase 3. In addition, the restoration of glutathione concentration suffered an intracellular depletion induced by H_2_O_2_. The authors suggested these phenolic compounds could be a therapeutic agent for the treatment or prevention of neurodegenerative diseases [153].

Izuta et al. [136] studied the effect of Chinese propolis components (among which AC is found) against tunicamycin-induced apoptosis in SH-SY5Y cells. The authors reported the inhibition of neuronal death, and that propolis decreased the induced activation of caspase 3 and the effects on mitochondrial membrane potential disruption [136]. This empirical data allows us to project a promising alternative to the current challenges faced by the aging human population and the appearance of neurodegenerative pathologies, such as AD.

#### 5.2.2. Anti-Neuroinflammatory Mechanisms

AD is also induced by neuroinflammation processes that occur as a brain immune barrier [117,154]. This immune response causes both an invasion of the glia by circulating immune cells and the production of pro-inflammatory cytokines, tumor necrosis factor (TNF)-α, interleukin (IL) IL-1β and IL-6-, nitric oxide (NO), prostaglandin E2 (PGE_2_), chemokines, and ROS. Glial cytokines can bind to specific receptors on the neuronal membrane and activate apoptotic pathways. TNF-α has been reported to bind to tumor necrosis factor (TNFR1), leading to neuronal apoptosis [155,156]. Reports show that bioactive molecules such as phenolic compounds, flavonoids and non-flavonoids, carotenoids, and others can exert neuroprotective effects by suppressing the activation of the microglia that modulates inflammatory processes in the CNS [119,157]. Although currently unclear, the proposed anti-inflammatory mechanisms of phenolic compounds at the brain level include:

(1) an inhibitory role in the release of cytokines, such as IL-1β and TNF-α, from activated glia;
(2) an inhibitory action against inducible nitric oxide synthase (iNOS) induction and subsequent NO production in response to glial activation;
(3) an ability to inhibit the activation of NADPH oxidase and subsequent generation of ROS in activated glia;
(4) an inhibitory activity regulation of pro-inflammatory transcription factors such as nuclear factor (NF)-κB through the modulation of various glial and neuronal signaling pathways [158,159]. Ferulic acid, CA, tyrosol, hydroxytyrosol, and vanillic acid have been evaluated as therapeutic agents in neuroinflammation diseases [160].

#### 5.2.3. Anti-Aggregation of β Amyloid Peptides

One of the main pathological characteristics of AD is the formation of amyloid plaques in the extracellular matrix of the cerebral cortex, caused by the overproduction of the Aβ peptide mediated by cholinergic cells. This phenomenon is caused by proteolytic cleavage of the APP and intracellular NFT [161]. Miyamae et al. [140] conducted studies in SH-SY5Y cells to evaluate the inhibition of the aggregation of the β-amyloid-42 protein. The results showed that CQA, in particular 4,5-di-O-caffeoylquinic acid and 3,4,5-tri-*O*-caffeoylquinic acid, strongly inhibited β-amyloid-42 aggregation, concluding that the caffeoyl group in CQA is essential for inhibitory activity in the neuroblastoma model. Recent reports point to the Aβ peptides production regulation, through enzyme inhibition associated with the amyloidogenic cascade, mainly β-secretase [162,163,164]. This protein known as the BACE performs proteolysis by generating a soluble fragment in the extracellular space known as “soluble APPβ” and an additional fragment bound to the plasma membrane, CTFβ peptide that undergoes proteolysis yet again by γ-secretase to generate toxic neuropeptides Aβ with 40–43 amino acids, finally causing the amyloid plaques characteristic of AD [165,166,167]. The use of bioactive molecules that inhibit the amyloidogenic pathway is a key alternative for the delay of AD in the early stages of the disease.

#### 5.2.4. Anticholinesterase Mechanisms

**Figure 1:**
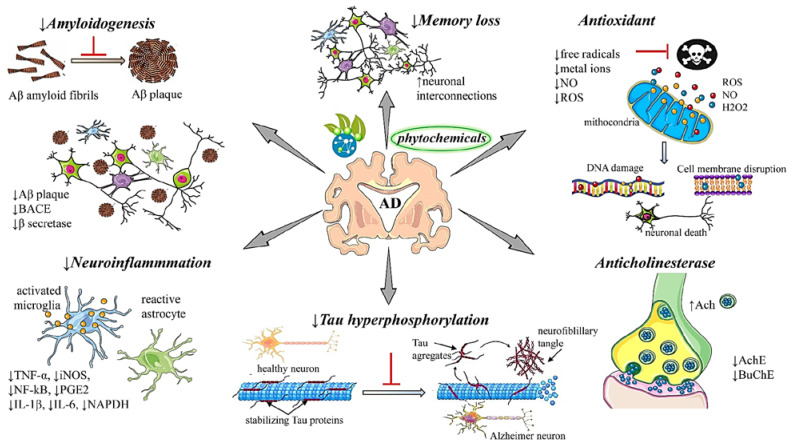
Summarized scheme with the most representative neuroprotective mechanisms of actions and effects of phytochemicals in Alzheimer’s disease. Abbreviations and symbols: ↑ increase, ↓ decrease, acetylcholine. (ACh), acetylcholinesterase (AchE), amyloid beta (Aβ), beta-site amyloid precursor protein cleaving enzyme 1 (BACE1), butyrylcholinesterase (BuChE), inducible nitric oxide synthase (iNOS), tumor necrosis factor (TNF)-α, interleukin (IL), nitric oxide (NO), nuclear factor kappa-light-chain-enhancer of activated B cells (NF-kB), Prostaglandin E_2_ (PGE2), nicotinamide adenine dinucleotide phosphate. (NADPH), reactive oxygen species (ROS), tumor necrosis factor-alpha (TNF-α).

Another key indicator in the triggering of AD is the decrease in the neurotransmitter ACh, due to AchE and BuChe (butyrylcholinesterase) increase. Studies have revealed that QA and chlorogenic acid extracted from plant matrices can inhibit the catalytic activities of these enzymes, avoiding the cholinergic deficits that occur in AD which trigger memory loss. Oboh et al. [150] evaluated the effect of the interaction between QA and CQA in murine models, using enzymes AChE and BuChE. Results showed that QA had greater inhibition potential than CQA, concluding that these bioactive molecules can exert neuroprotective properties through the inhibition of AchE and BuChE, suggesting a possible mechanism that slows the ACh and butyrylcholine hydrolysis in the brain [150]. Arya et al. [168] carried out an analysis of lipidiol and amberboin sesquiterpenes belonging to an important category of terpenoids found mainly in herbaceous plants and capable of causing inhibition in cholinesterases with an IC_50_ < 9 M, being potential candidates for learning and memory improvement in patients with AD, and possible preventive treatment for this neurodegenerative pathology. Table 2 and Figure 1 presents the effects of the main phytochemicals described in the literature with compounds capable of having an antioxidant, anti-cholinesterase, anti-amyloidogenic, and anti-neuroinflammation effect.

## 6. Phytochemicals in AD: From Bioactive Effects to Bioavailability Issues

Phytochemicals’ activity against AD occurred through different mechanisms such as their anti-amyloid, anticholinesterase, anti-inflammatory, and antioxidant properties [198]. The worldwide increasing tendency toward using phytochemicals may be due to low toxicity, and synergistic effects [199,200,201]. Bioavailability is a crucial aspect of pharmacokinetics that reflects the quantity of a specific compound given by different routes that reach the site of action [201,202,203]. The shortness of current agents used against AD can be attributed to several factors and low bioavailability is considered one of the most important ones among them [204]. How fast the phytochemicals will be absorbed after administration is answered by bioavailability. The complex active ingredient of plants makes this field of research more and more difficult to be investigated [205,206]. One of the significant challenges for the pharmacological reach in the central nervous system (CNS) is the blood-brain barrier permeability [207]. A high percentage of phytochemicals with high molecular weight and a high degree of lipophilicity will not be able to cross into the brain compartment, combined with chemical instability in the bloodstream [208]. For this reason, incorporating bioactive molecules into nanoparticles is a strategy that allows for overcoming various physicochemical limitations of the drug [203,209]. However, phytochemicals rarely fit in the ideal drug-like profile due to their scarce bioavailability; therefore, their manipulation also aims to ameliorate their pharmacokinetics [210,211]. To reach this scope, other approaches could be adopted, such as the application of drug delivery systems (DDS) [212]. After several decades of research in nanotechnology to transport drugs to the CNS, it is possible to have nanoparticles that allow an adequate transport of phytochemicals in a targeted way and with a control in the release time [213,214]. Mainly due to a sophisticated surface modification of the nanoparticle that allows adequate transit to the brain [215,216]. In the last decade, micro and nanospheres, polymeric micelles, nanocapsules and, in general, nanoparticulate systems have been shown to have an effect in improving the specific targeting of bioactive compounds, decreasing their systemic toxicity, improving the therapeutic efficiency rate and protecting active substances against biochemical degradation [217,218].

The low solubility of α-bisabolol, isolated from *Matricaria chamomilla* L. essential oil leads to significantly reduced bioavailability [219]. Poor bioavailability it’s the reason for too low brain uptake of curcumin [220]. Phytol is found in nature and exerts a wide range of biological effects, but low absorption and poor solubility lead to reduced bioavailability which hinders the clinical use of this compound [221]. The reason that scientists cannot replicate the beneficial effects of resveratrol against AD in humans can be its low oral bioavailability [222,223]. These examples of scientific studies showed low bioavailability as an obstacle in the way of beneficial activity of phytochemicals. The limitations in bioavailability issue, which can be hindrance in the use of phytochemicals against to AD, can be overcome through delivery methods, such as cyclodextrins, implants, nanoparticles, niosomes, and liposomes. A very popular strategy in designing new anti-AD agents aims to modify natural bioactive compounds, whose polypharmacology has been intensively investigated over the years. Many naturally occurring substances such as resveratrol, neoechinulins, genistein or diosgenin provided antioxidant, anti-inflammatory, chelating, and/or scavenger activity [115,200,224,225]. Therefore, the conjunction with other pharmacophores, such as moiety belonging to an already in-use anti-AD drug, creates a new molecular entity possessing an endowed spectrum of activities [24].

## 7. Overall Conclusions

In the coming decades, there will be a considerable increase in neurodegenerative diseases, including Alzheimer’s disease. For this reason, research and biotechnological progress in new strategies for both prevention and treatment are essential. Currently, the approach to therapy against AD is based on inhibiting one pathophysiological mechanism at the same time, a role played by the four FDA-approved drugs, including AChEIs and NMDA receptor agonists. However, according to previous studies, these drugs maintain maximum effectiveness three years after administration, causing advancement of the pathology after this time. The need arises to find new drugs that achieve the objective of preventing and treating AD, being the use of phytochemicals an attractive approach. Phytochemicals have antioxidant, anti-inflammatory, anti-amyloid, and anticholinesterase properties, which are the four fundamental pillars identified in the pathophysiological process of Alzheimer’s. It has been shown that various components of plants, such as phenolic compounds, flavonoids and non-flavonoids and/or carotenoids, have neuroprotective effects, which not only act at the level of blocking the progression of AD but also have beneficial properties on AD prevention. This is because these phytopharmaceuticals act in a broad spectrum, inhibiting various mechanisms that trigger or progress the disease. However, there is a limitation in the use of phytopharmaceuticals and this corresponds to their bioavailability, a crucial aspect in terms of pharmacokinetics, but which can be overcome through the use of administration methods such as cyclodextrins, implants, nanoparticles, niosomes or liposomes. From a future perspective, overcoming the low bioavailability of phytochemicals is crucial and biotechnological tools will be required, as well as new clinical studies using phytochemicals and derivatives against AD. The study of new drugs for the prevention and/or treatment of AD is a great challenge for clinical research, which must be promoted to provide better well-being and a higher quality of life for people who suffer from these neurodegenerative diseases.

## Figures and Tables

**Table 1:** Summarized data regarding the candidate vaccines for the potential treatment of AD.

Target of the Candidate Vaccine in AD	Example/Manufacturer	Results	Trial Status	Ref
β-amyloid Vaccines	UB-311 Vaxxinity	improve cognitive function	Phase 2	[51,52]
	ABvac 40 Araclon Biotech’s			[50]
	ACI-24 AC Immune	increase clearance of β-amyloid plaques		[49]
Tau Vaccines	ACI-35.030 AC Immune	no efficacy on tau increase the immune response	Phase 2	[53]
	AADvac1 Axon Neuroscience	improve cognitive function		[54]
Immunomodulatory Vaccines	GV1001 GemVax KAEL Bio	increase clearance of β-amyloid plaques and tau tangles anti-neuroinflammatory	Phase 2	[55]
	Bacillus Calmette-Guerin Mindful Diagnostics and Therapeutics	nasal administration impact on β-amyloid plaques immunostimulatory		[56]
	Protollin Brigham and Women’s Hospital	increase immune response	Phase 1	[48]

**Table 2:** Summarized data regarding pharmacological effects of phytochemicals as potential agents in AD management.

Tested Phytochemical	Plant Origin	Experimental Model	Effects	Ref.
Anthocyanin 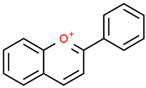	*Phaseolus vulgaris* L. Korean Black Beans *Morus alba* L. (Mulberry) *Vaccinium myrtillus* L. (Blueberry)	In vivo APP/PS1 mice In vitro murine model SAMP8 SAMR1 Mouse hippocampal cells HT22 Murine model Neuroblastoma cells	Antioxidant Anti-amyloidogenic	[169,170,171]
Curcumin 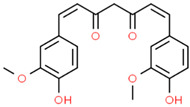	*Curcuma longa* L. (Turmeric)	In vivo Alzheimer-Pathological Model of *Caenorhabditis elegans* In vitro Post-Mortem Brain Tissue Murine model	Antioxidant Anti-amyloidogenic	[172,173,174,175]
Resveratrol 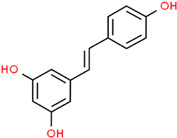	*Vitis vinífera* L. (seeds) *Veratrum grandiflorum* (Maxim. ex Miq.) O.Loes. *Vaccinium myrtillus* L. *Fragaria sp.* *Rubus ideaeus* L.	In vitro Murine macrophages RAW 264.7 Microglia Munira BV-2 Murine models SH-5ySy	Antioxidant Anti-amyloidogenic Anti-hyperphosphorylation Anti neuro-inflammatory	[176,177,178,179]
Berberin 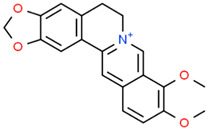	*Berberis* sp.	In vitro Neuroblastoma 2a Murine model In vivo Transgenic mice model	Antioxidant Anti-amyloidogenic Anti neuro-inflammatory Tau Anti-hyperphosphorylation	[180,181,182]
Galantamine 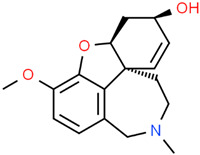	*Galanthus alpinus* Sosn. *Galanthus woronowii* Losinsk. *Lycoris radiata* (L’Hér.) Herb.	In vitro APPswe/PS1dE9 murine model Brain microvasculating endothelial cells HEK293 (APP-HEK293) Neuroblastoma SH-5ySy cells	Anticholinesterase Anti-amyloidogenic Anti-neuroinflammatory	[64,164,178,183,184,185,186]
Terpenoid 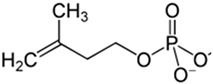	*Ginkgo biloba* L. *Elettaria cardamomum* (L.) *Maton* *Pueraria lobata* *(Willd.) Ohwi.* *Ganoderma* sp.	In vitro Murine model BACE enzyme acetylcholinesterase SH-5ySy cells Fibroblasts GMK cells	Anticholinesterase Anti-amyloidogenic Antioxidant Anti-neuroinflammatory	[168,187,188,189,190,191]
Catechin 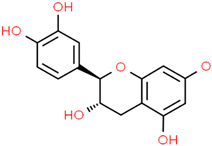	*Rhizophora mucronata Lam.* *Camellia sinensis* (L.) Kuntze	In vitro PC12 cells Acetylcholinesterase enzyme Murine model	Anticholinesterase Anti-amyloidogenic Antioxidant Anti-neuroinflammatory	[192,193,194,195]
Hydroxycinnamic acids caffeoylquinic acid 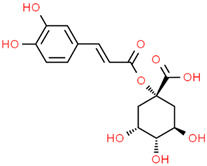 chlorogenic acid 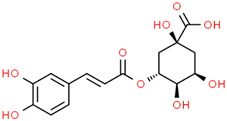 caffeic acid 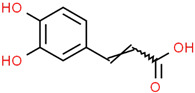 ferulic acid 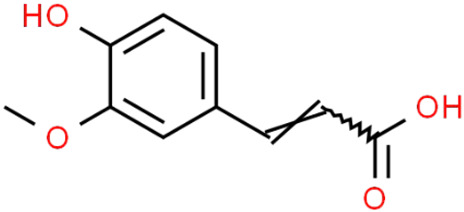	*Coffee grounds* (*Coffea arabica* L.) *Green coffee beans* (*Coffea arabica* L.) *Roasted coffee beans* (*Coffea arabica* L.)	In vitro SH-5ySy cells Neuro-2A neuroblastoma PC12 cells HepG2 Primary culture cortical Neurons Murine models In vivo *Drosophila* (Alzheimer’s) models	Anticholinesterase Anti-amyloidogenic Antioxidant Anti -neuroinflammatory Tau Anti-hyperphosphorylation	[144,145,147,148,150,196,197]

## Data Availability

Not applicable.
